# Supplementary material for: Splice-Junction-Based Mapping of Alternative Isoforms in the Human Proteome
Source: Cell Rep. Author manuscript; Available in PMC 2020 Jan 15. (PMC6961840; doi:10.1016/j.celrep.2019.11.026)
Supplement: 3 [file NIHMS1546469-supplement-3.zip › DF2/PXD0010154/Prostate-61-Q8IZE3-NTDLSLEGDPFSQPIKFPINGLSDVK.pdf]

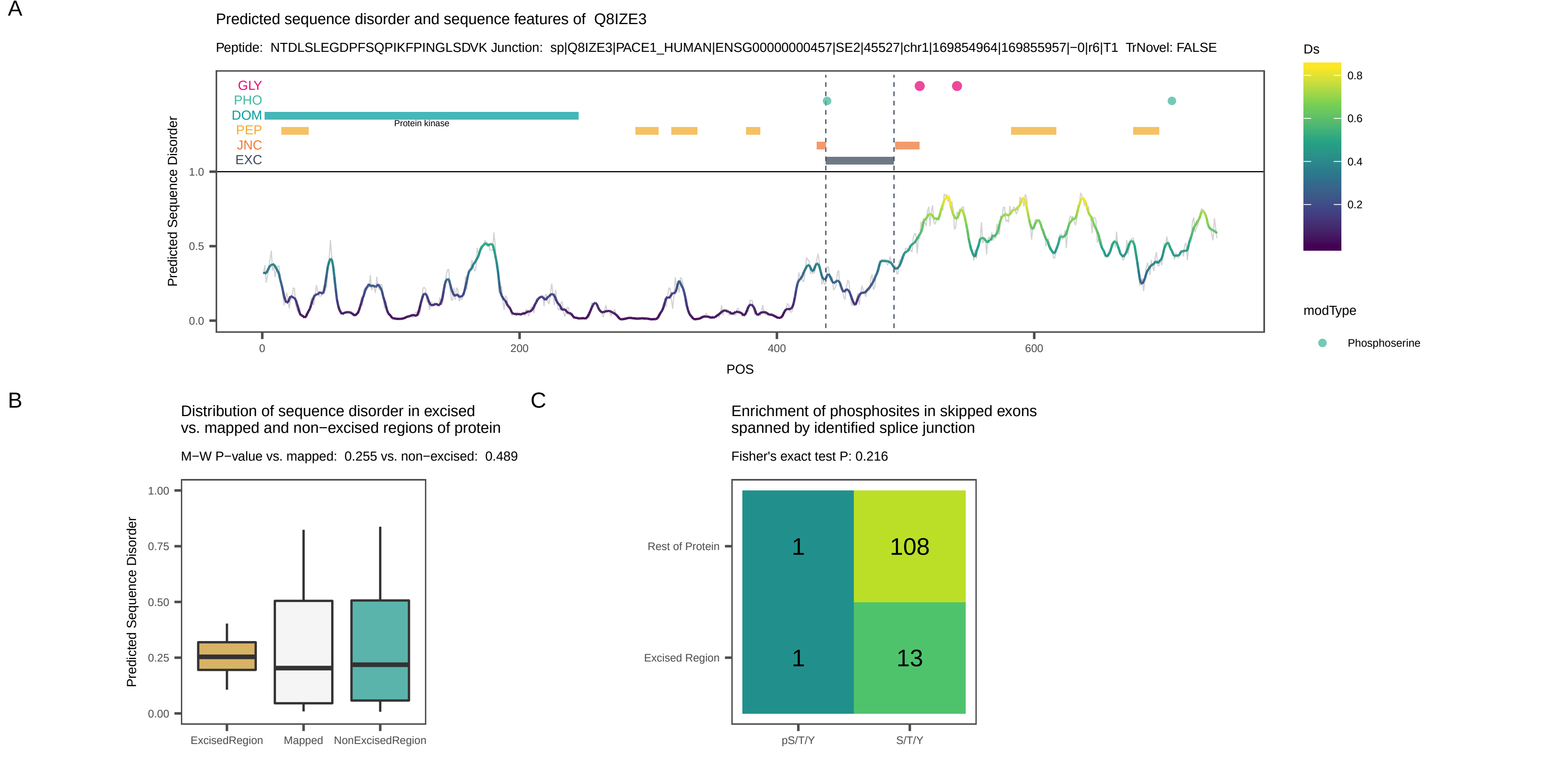

A
Predicted sequence disorder and sequence features of Q8IZE3
Peptide: NTDLSLEGDPFSQPIKFPINGLSDVK Junction: sp|Q8IZE3|PACE1_HUMAN|ENSG00000000457|SE2|45527|chr1|169854964|169855957|−0|r6|T1 TrNovel: FALSE
GLY
PHO
DOM
PEP
JNC
EXC
Protein kinase
Predicted Sequence Disorder
1.0
0.5
0.0
0
200
400
600
POS
Ds
0.8
0.6
0.4
0.2
modType
Phosphoserine
B
Distribution of sequence disorder in excised
vs. mapped and non−excised regions of protein
M−W P−value vs. mapped: 0.255 vs. non−excised: 0.489
Predicted Sequence Disorder
1.00
0.75
0.50
0.25
0.00
ExcisedRegion
Mapped
NonExcisedRegion
C
Enrichment of phosphosites in skipped exons
spanned by identified splice junction
Fisher's exact test P: 0.216
Rest of Protein
Excised Region
1
108
1
13
pS/T/Y
S/T/Y
